# Supplementary figures and images for: A novel machine learning derived RNA-binding protein gene–based score system predicts prognosis of hepatocellular carcinoma patients
Source: PeerJ. 2021 Dec 20;9:e12572. doi: 10.7717/peerj.12572 (PMC8697767; doi:10.7717/peerj.12572)

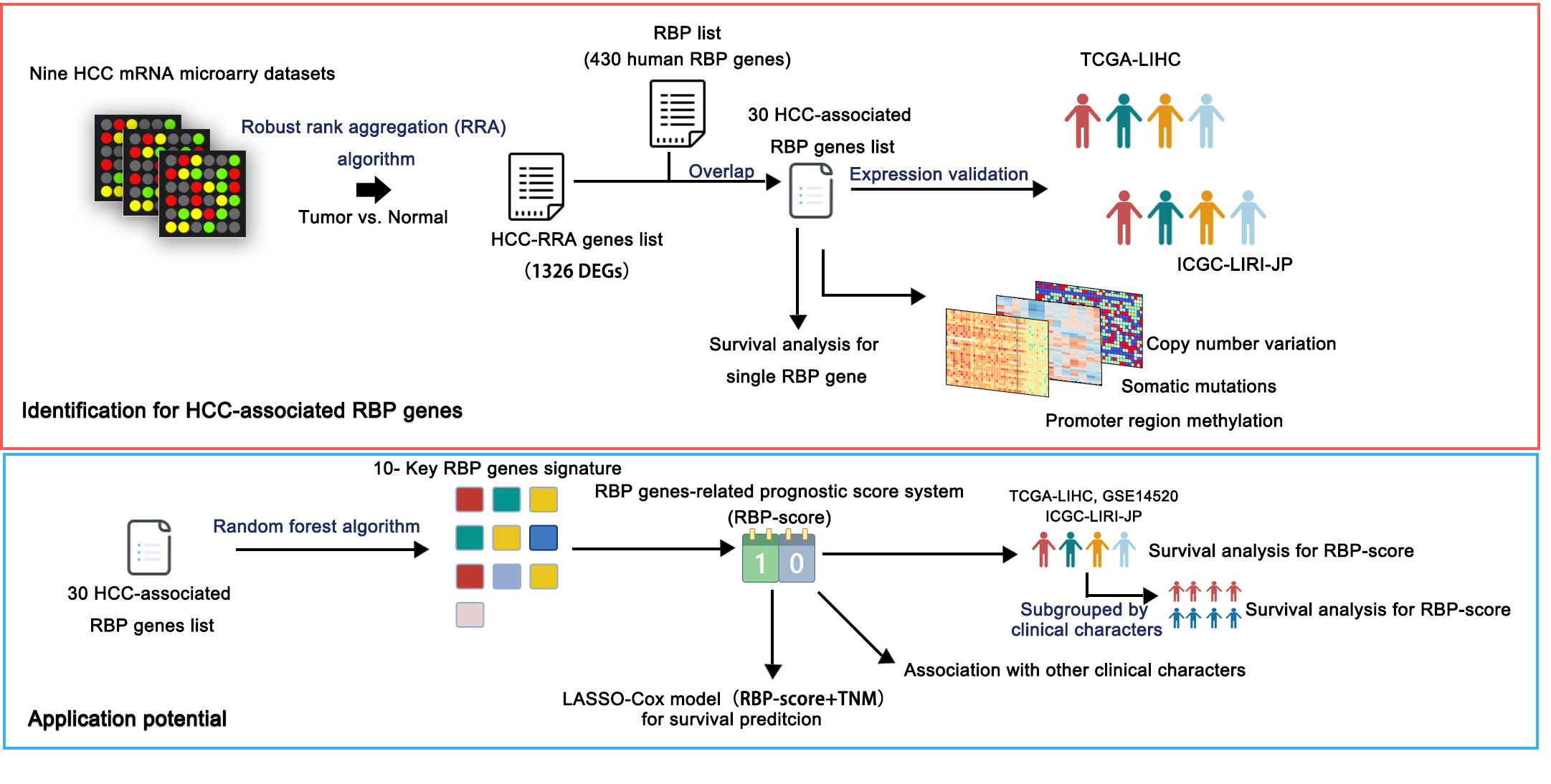

Supplement: Supplemental Information 1 [file peerj-09-12572-s001.jpg]

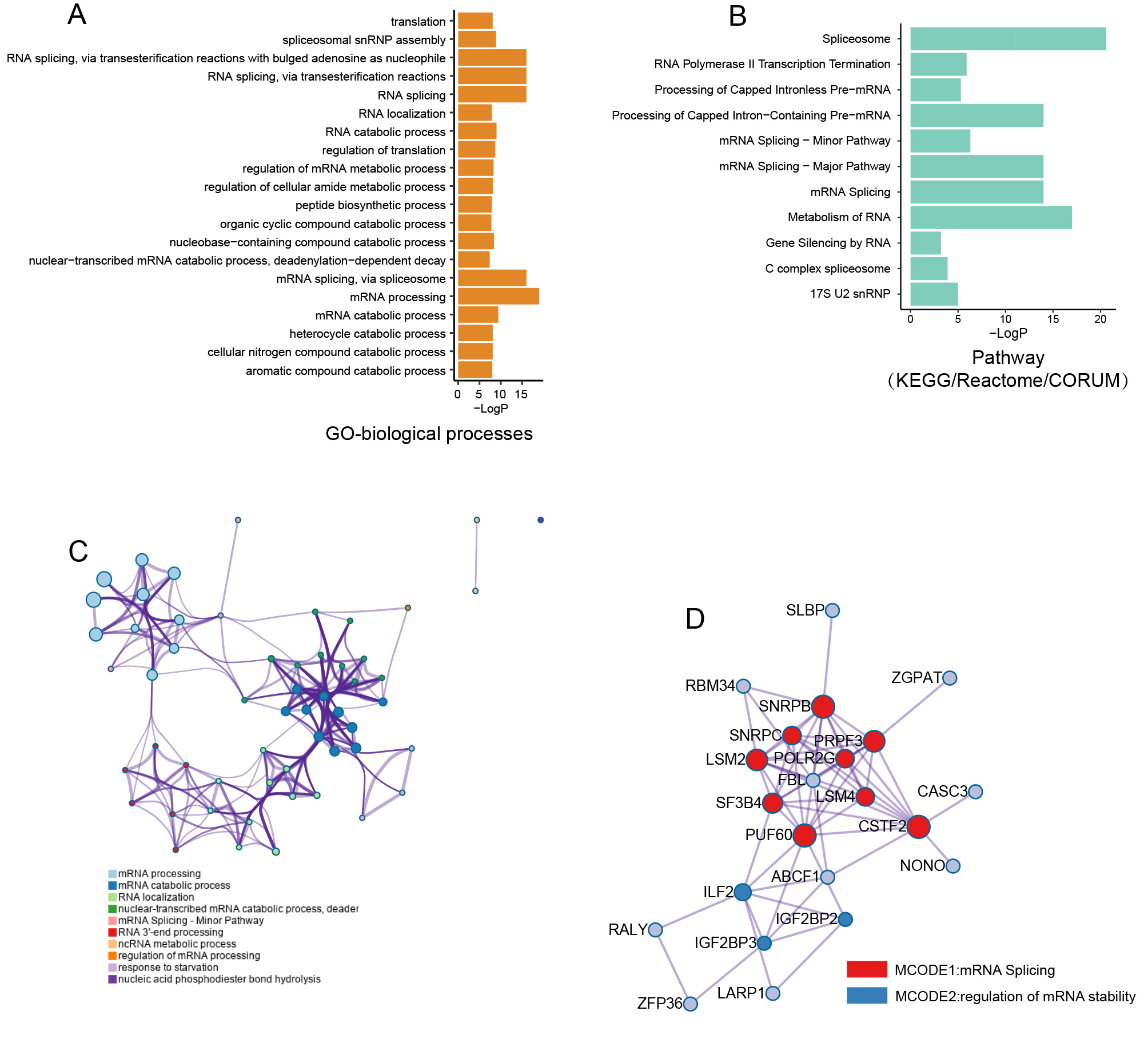

Supplement: Supplemental Information 2 — (A) The top 20 (sorted by P value) from enrichment analysis result for GO biological processes.(B) the top 20 (sorted by P value) from enrichment analysis result for pathways; (C) A clustering network formed by correlated terms from the functional enrichment analysis; (D) Protein-protein interaction (PPI) enrichment network for production of thirty RBP genes was built by molecular complex detection (MCODE) algorithm. [file peerj-09-12572-s002.jpg]

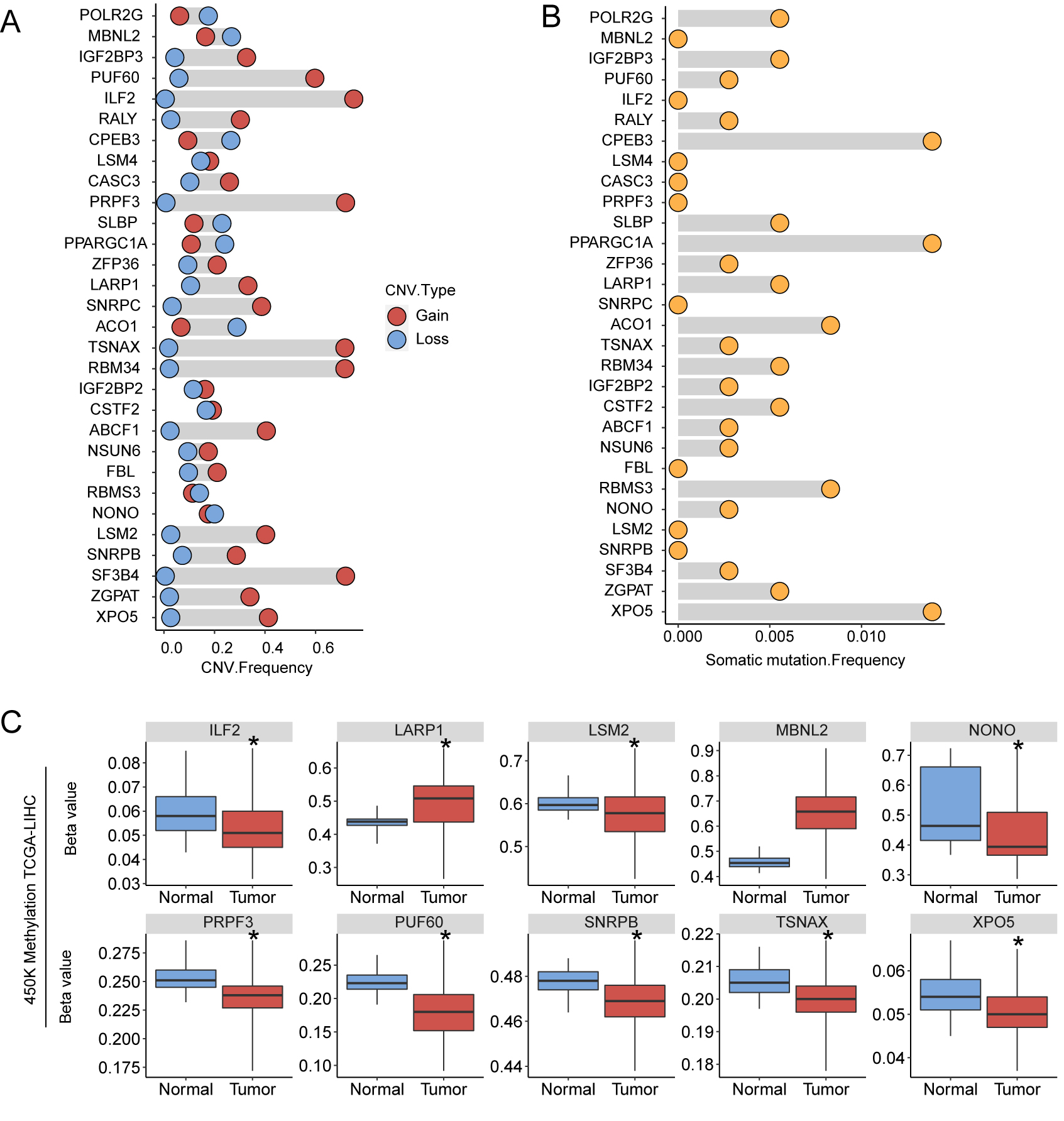

Supplement: Supplemental Information 3 — (A) Copy number variation of 30 HCC-associated RBP genes in TCGA-LIHC; (B) Somatic mutation frequency of thirty HCC-associated RBP genes; (C) Significant promoter-region methylation level changes were detected using 450K Methylation microarry in TCGA-LIHC. *P < 0.05 compared with Normal tissue. [file peerj-09-12572-s003.jpg]

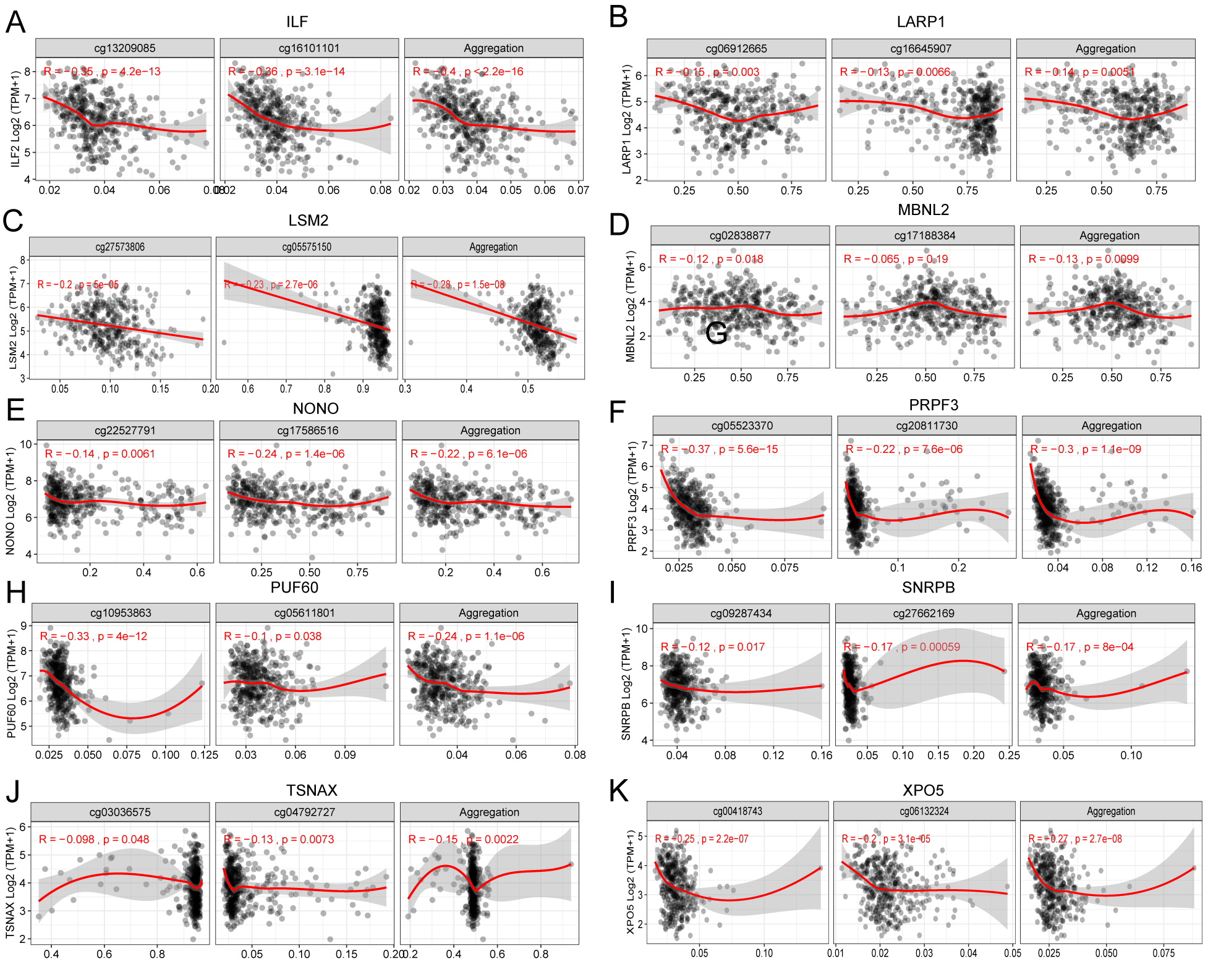

Supplement: Supplemental Information 4 [file peerj-09-12572-s004.jpg]

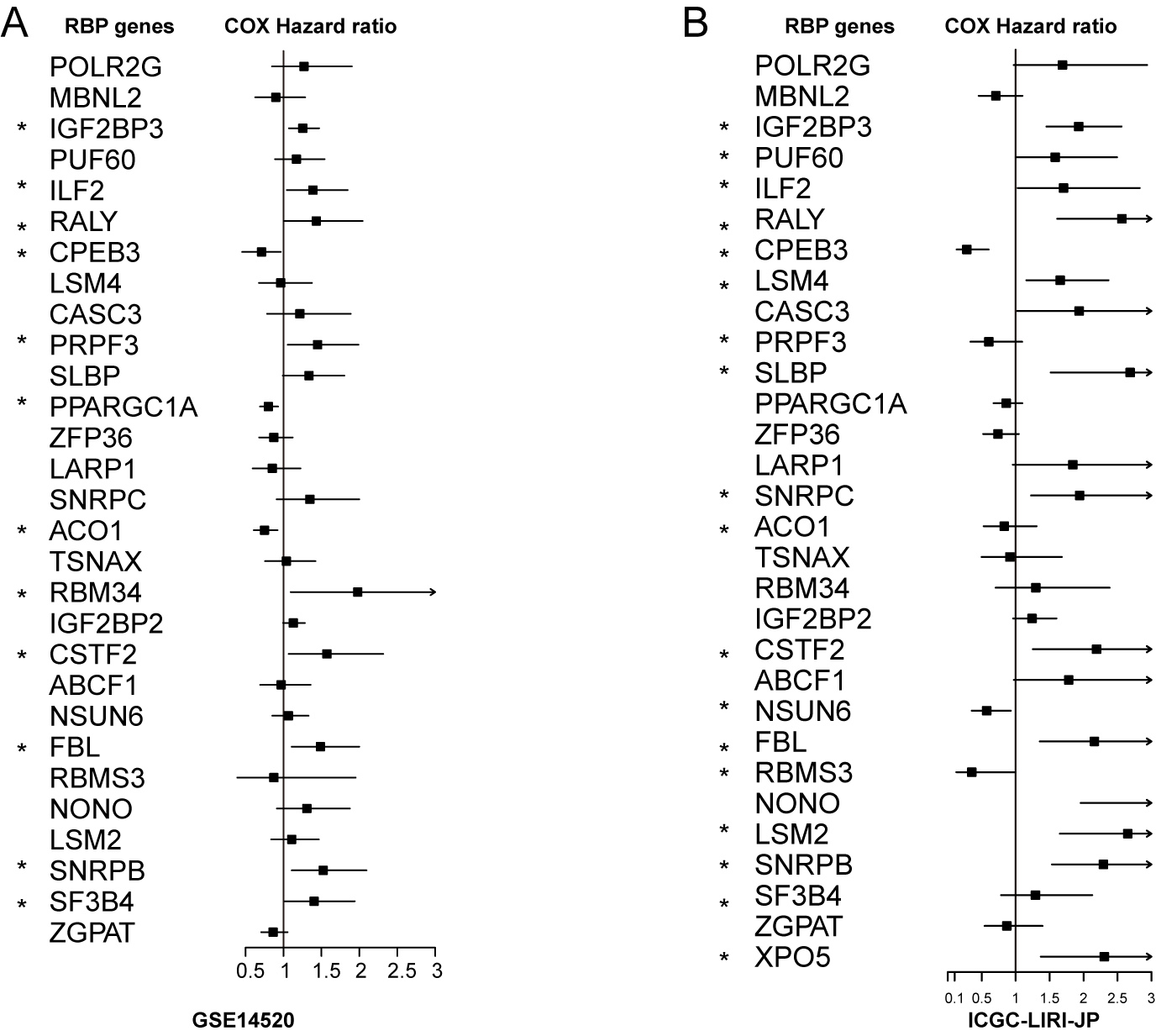

Supplement: Supplemental Information 5 — (A) and ICGC-LIRI-JP (B) were calculated by Cox model. [file peerj-09-12572-s005.jpg]

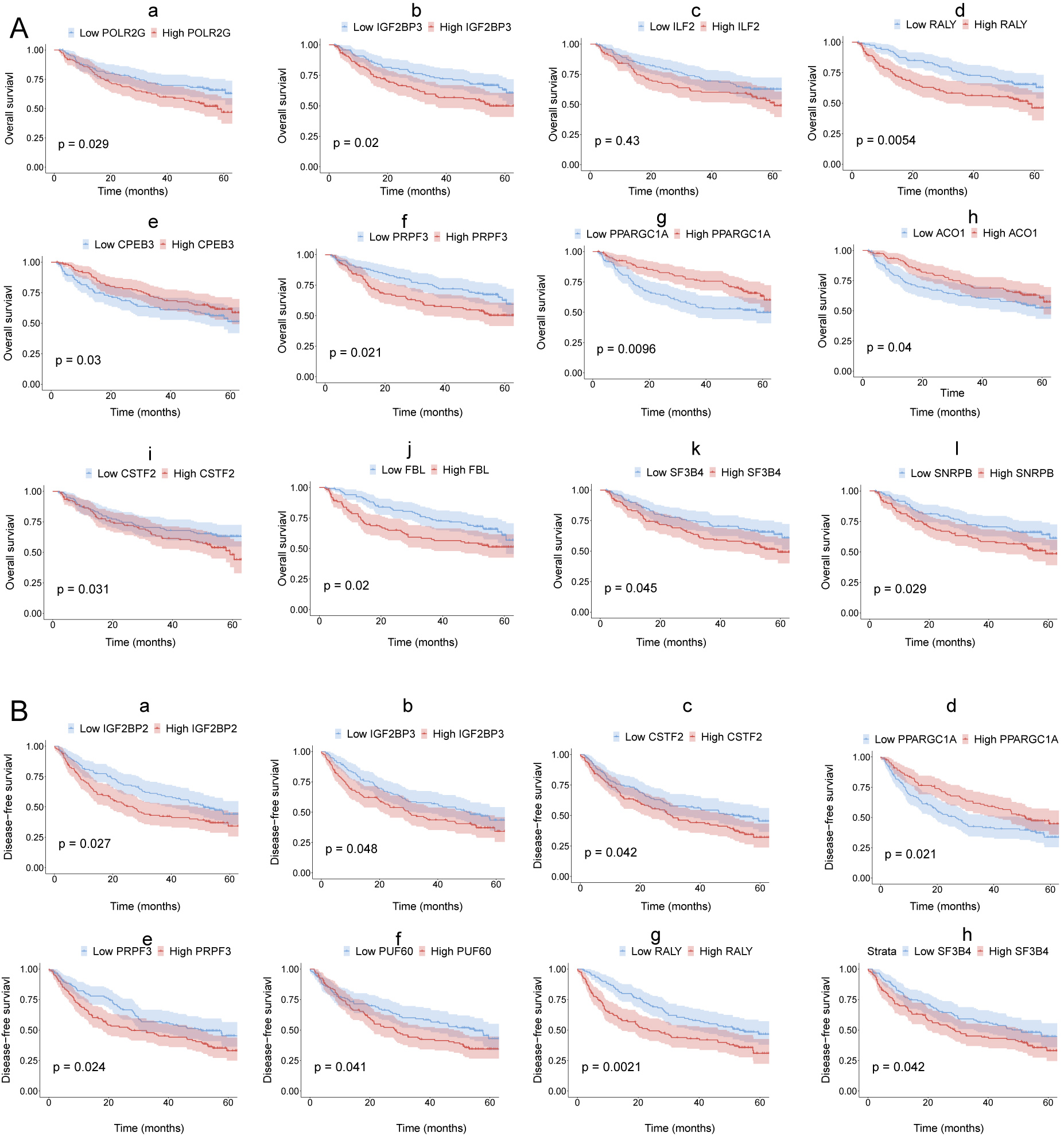

Supplement: Supplemental Information 6 — Kaplan–Meier curves of RBP genes that associated overall survival (A) and disesase-free survival (B) in GSE14520. [file peerj-09-12572-s006.jpg]

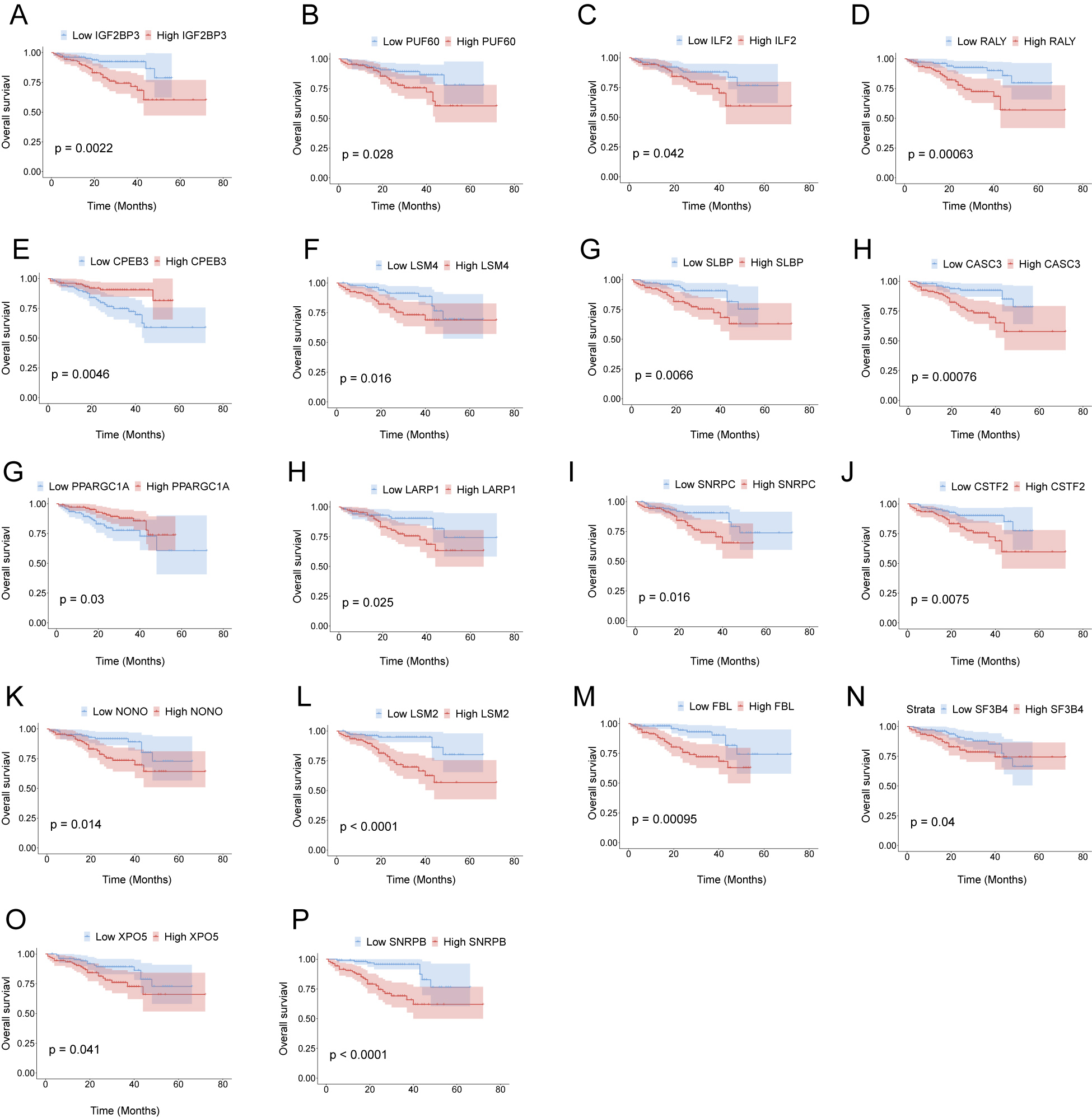

Supplement: Supplemental Information 7 [file peerj-09-12572-s007.jpg]

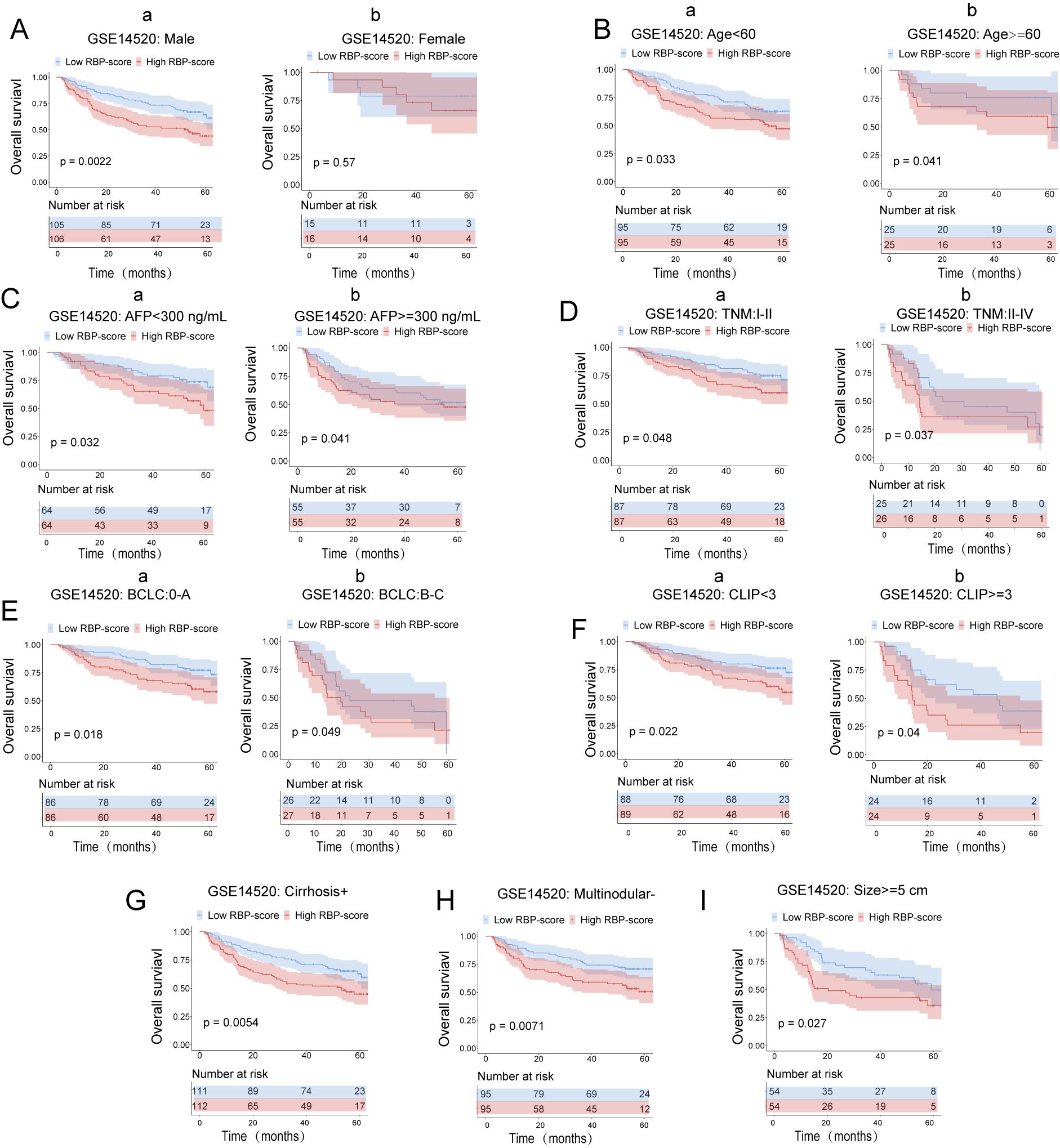

Supplement: Supplemental Information 8 — In GSE14520 patients were stratified by gender (A), age (B), AFP level (C), TNM staging (D), BCLC staging (E), CLIP staging (F), cirrhosis condition (G),multinodular state (H) and tumor size (I). The overall survival of patients with different RBP-score in each subgroup were compared. [file peerj-09-12572-s008.jpg]

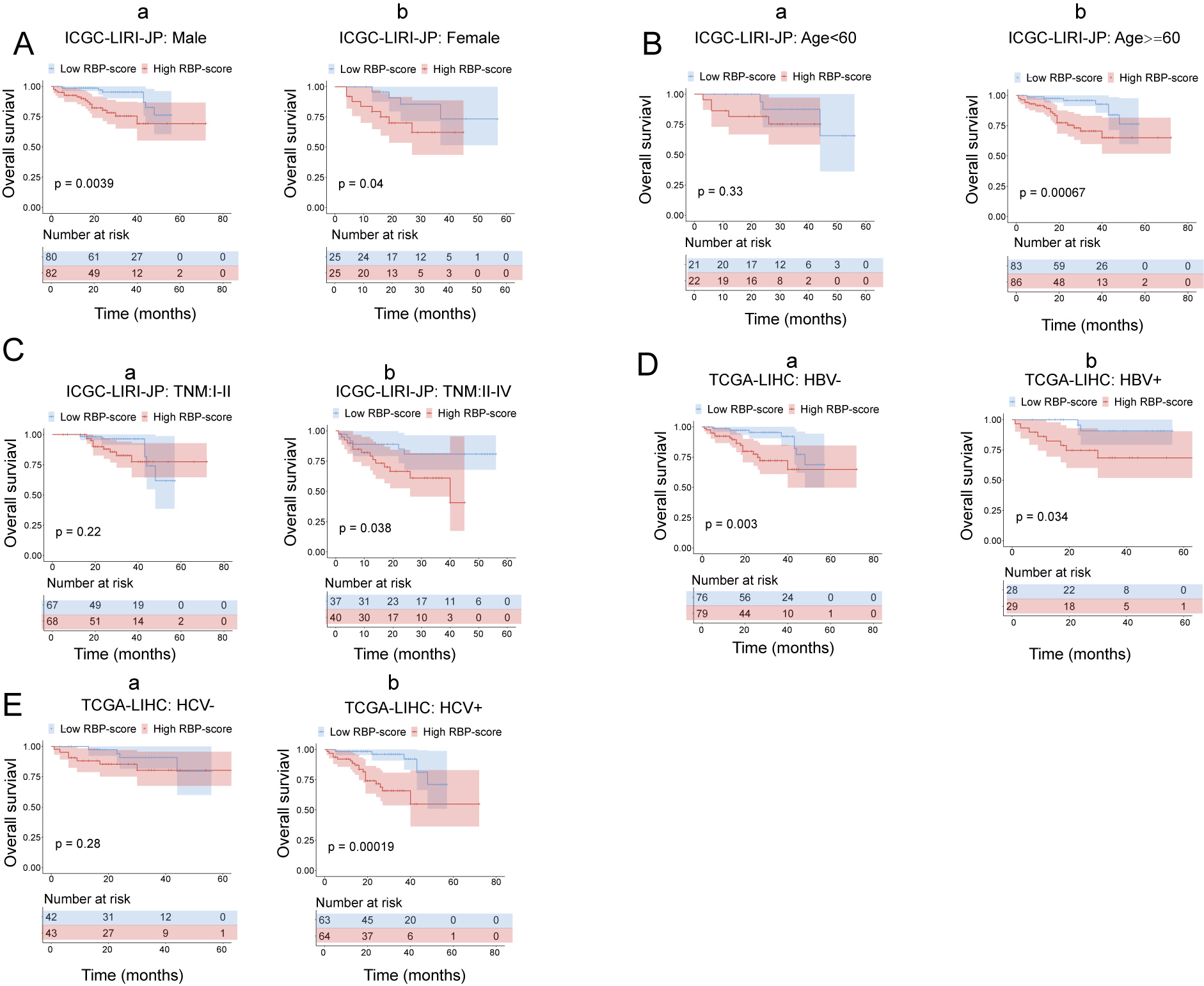

Supplement: Supplemental Information 9 — In ICGC-LIRI-JP patients were stratified by gender (A), age (B), AFP level (C), TNM staging (C), HBV infection (D) and HCV infection (E). The overall survival of patients with different RBP-score in each subgroup were compared. [file peerj-09-12572-s009.jpg]

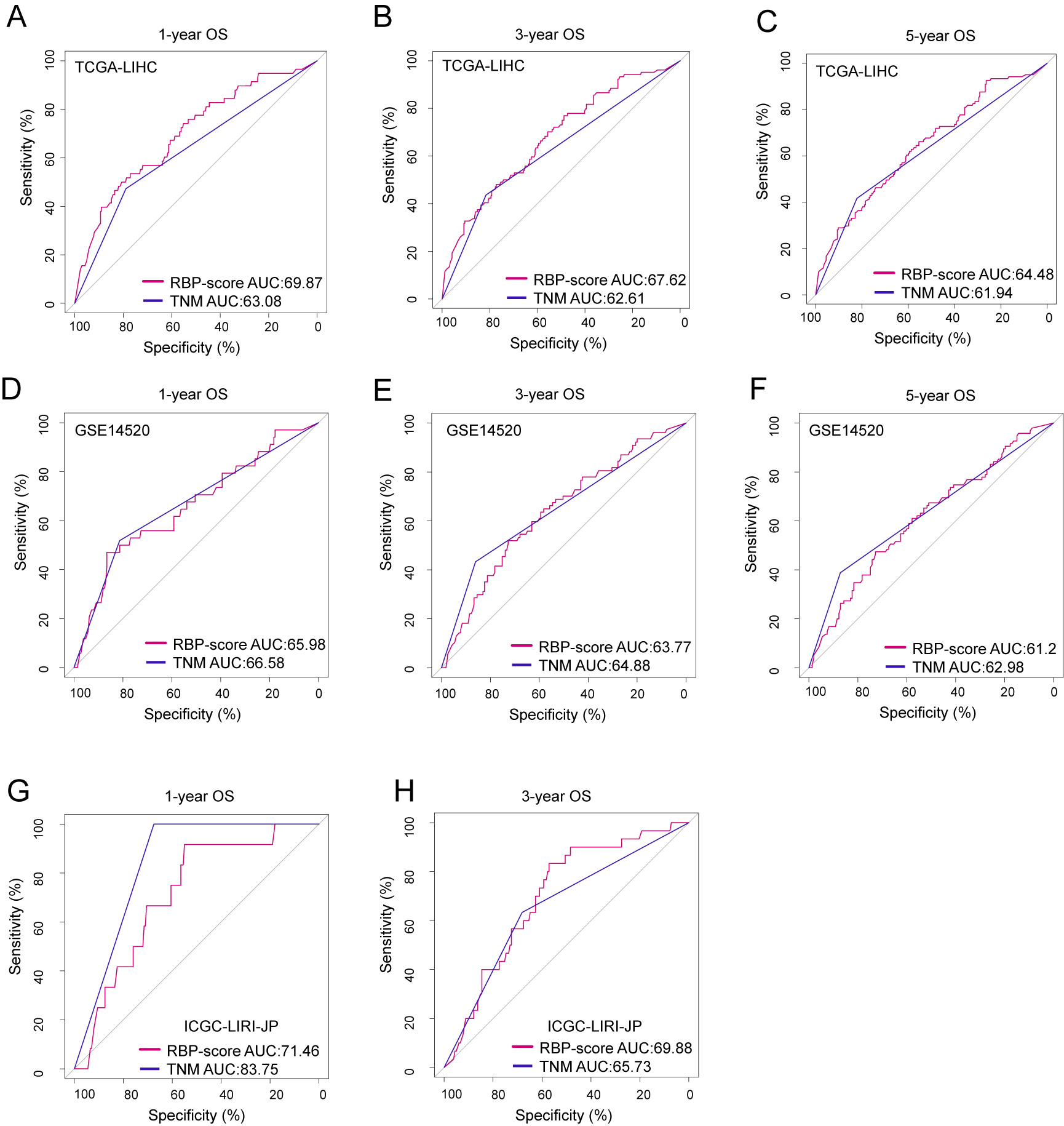

Supplement: Supplemental Information 10 — ROC curves were used to indicate the accuracy of RBP score and TNM stageing to estimate 1-year, 3-year and 5-year overall survival patients from TCGA-LIHC (A–C), GSE1452 (D–F) and ICGC-LIRI-JP (G–H). [file peerj-09-12572-s010.jpg]
