# Supplementary material for: A novel machine learning derived RNA-binding protein gene–based score system predicts prognosis of hepatocellular carcinoma patients
Source: PeerJ. 2021 Dec 20;9:e12572. doi: 10.7717/peerj.12572 (PMC8697767; doi:10.7717/peerj.12572)
Supplement: Supplemental Information 11 [file peerj-09-12572-s011.docx]

Table S1 Information for datasets used in the present study

| Datesets | Type | sample size | | Gender | | Age | | TNM | |
| --- | --- | --- | --- | --- | --- | --- | --- | --- | --- |
|  |  | Tumor | Non-tumor | Male | Female | <=60 | >60 | Ⅰ-Ⅱ | Ⅲ-Ⅳ |
| GSE14520 | microarray | 242 | 239 | 211 | 31 | 196 | 46 | 139 | 103 |
| GSE22058 | microarray | 100 | 97 | N/A | N/A | N/A | N/A | N/A | N/A |
| GSE25097 | microarray | 268 | 243 | N/A | N/A | N/A | N/A | N/A | N/A |
| GSE36376 | microarray | 240 | 193 | N/A | N/A | N/A | N/A | N/A | N/A |
| GSE45436 | microarray | 95 | 39 | N/A | N/A | 79 | 16 | N/A | N/A |
| GSE64041 | microarray | 60 | 60 | N/A | N/A | N/A | N/A | N/A | N/A |
| GSE76427 | microarray | 115 | 52 | 93 | 22 | 48 | 67 | 90 | 25 |
| GSE54236 | microarray | 81 | 80 | 64 | 17 | N/A | N/A | N/A | N/A |
| GSE63898 | microarray | 228 | 168 | N/A | N/A | N/A | N/A | N/A | N/A |
| TCGA-LIHC | RNA-seq | 356 | 49 | 234 | 122 | 170 | 186 | 262 | 94 |
| ICGC-LIRI-JP | RNA-seq | 212 | 177 | 153 | 59 | 43 | 169 | 129 | 83 |
